# Microbial Translocation and Perinatal Asphyxia/Hypoxia: A Systematic Review

**DOI:** 10.3390/diagnostics12010214

**Published:** 2022-01-16

**Authors:** Dimitra-Ifigeneia Matara, Abraham Pouliakis, Theodoros Xanthos, Rozeta Sokou, Georgios Kafalidis, Zoi Iliodromiti, Theodora Boutsikou, Nicoletta Iacovidou, Christos Salakos

**Affiliations:** 1 Neonatal Department, Medical School, Aretaieio Hospital, National and Kapodistrian University of Athens, 11528 Athens, Greece; kontifi@gmail.com (D.-I.M.); gkafalidis@gmail.com (G.K.); ziliodromiti@yahoo.gr (Z.I.); theobtsk@gmail.com (T.B.); niciac58@gmail.com (N.I.); 2 2nd Department of Pathology, “Attikon” Hospital, Medical School, National and Kapodistrian University of Athens, 12462 Athens, Greece; apouliak@med.uoa.gr; 3 Medical School, European University Cyprus, Nicosia 2404, Cyprus; theodorosxanthos@yahoo.com; 4 Pediatric Surgical Department, “Attikon” Hospital, Medical School, National and Kapodistrian University of Athens, 12462 Athens, Greece; csalakos@otenet.gr

**Keywords:** microbiome, gut microbiota, microbial translocation, perinatal asphyxia, endotoxin, lipopolysaccharides, animal model

## Abstract

The microbiome is vital for the proper function of the gastrointestinal tract (GIT) and the maintenance of overall wellbeing. Gut ischemia may lead to disruption of the intestinal mucosal barrier, resulting in bacterial translocation. In this systematic review, according to PRISMA (Preferred Reporting Items for Systematic Review and Meta-Analysis) guidelines, we constructed a search query using the PICOT (Patient, Intervention, Comparison, Outcome, Time) framework. Eligible studies reported in PubMed, up to April 2021 were selected, from which, 57 publications’ data were included. According to these, escape of intraluminal potentially harmful factors into the systemic circulation and their transmission to distant organs and tissues, in utero, at birth, or immediately after, can be caused by reduced blood oxygenation. Various factors are involved in this situation. The GIT is a target organ, with high sensitivity to ischemia–hypoxia, and even short periods of ischemia may cause significant local tissue damage. Fetal hypoxia and perinatal asphyxia reduce bowel motility, especially in preterm neonates. Despite the fact that microbiome arouse the interest of scientists in recent decades, the pathophysiologic patterns which mediate in perinatal hypoxia/asphyxia conditions and gut function have not yet been well understood.

## 1. Introduction

The human body hosts about 100 trillion microorganisms, called microbiota, which are involved in multiple functions, such as vitamin synthesis, bile salt metabolism, fiber, mucus and fatty acid catabolism, regulation of inflammation and homeostasis of the immune system [1]. They colonize the skin, the mammary glands, the saliva and oral mucosa, the conjunctiva, the airway, the urogenital system and the GIT. The genome of the microbiota is called the microbiome and it has special characteristics, such as its own weight, genetic and cellular content and its own metabolic activity [1,2]. It begins to be established in utero by the maternal microbial flora. Microbial communities are isolated in healthy embryos in the uterus, umbilical cord blood, amniotic fluid, placenta, and embryonic membranes. The microbiome in neonates varies in composition and diversity, taking the adult type at 3–5 years of age [2,3,4].

In a healthy host, the microbiota plays a vital role in maintaining proper bowel function and good health in general. Changes in the microbiota’s composition can adversely affect the normal immune and metabolic pathways and the general well-being of the host. In other words, there is a dynamic relationship that can be transformed from ‘symbiotic’ to ‘dysbiotic’ and life-threatening [5,6,7]. Many factors are causally related to these fundamental changes such as genetic background, maternal diet prior to and during pregnancy, maternal BMI during pregnancy, the adequacy of nutrients’ intake, maternal microbiome, administration of probiotics, mode of delivery, gestational age, perinatal stress, administration of antibiotics to the mother during pregnancy and to the baby early in neonatal life, infections during pregnancy and during the perinatal or neonatal period, the newborn’s diet (breastfeeding, cow’s milk, mixed diet), and finally other environmental factors such as temperature, humidity, pH and oxygen levels in the tissues [8,9,10].

Intestinal mucosa acts as an anatomical and functional barrier (IMB) and plays an important role in preventing the leak of intestinal bacteria and their breakdown products to extra-enteric tissues. In pathological conditions, potentially harmful intraluminal agents escape to the mesenteric lymph nodes and to nearby and distant organs and tissues, and this results in dysfunction of many organs and even death. This phenomenon is called bacterial translocation [11,12,13].

The aim of this study is to systematically investigate whether asphyxia contributes to microbial translocation in neonates.

## 2. Materials and Methods

### 2.1. Study Design

This study was performed following the PRISMA (Preferred Reporting Items for Systematic reviews and Meta-Analyses) guidelines [14]. Eligible studies reported in PubMed, up to the study data collection time point (April 2021) were selected as being potentially eligible to be included. Only studies published in the English language were selected and there was a restriction on publication year, i.e., only publications after 1 January 1990 were requested; moreover no restrictions on publication type were posed. This was due to the lack of relevant information from previous years, as it is a field that has aroused the interest of scientists only in recent decades.

### 2.2. Search Question Formation

The search query was structured according to the PICOT (Population-Intervention-Comparison-Outcome-Time) framework [15]. After defining and joining PICO individual parts, the final question was issued in PubMed. The individual PICO components are depicted in Table 1; note that MeSH (Medical Subject Headings) terms were not extensively used and instead keywords were searched in abstract and title, as many publications do not use defined and appropriate MeSH terms.

Structured query took place according to the PICO framework. The final query issued in PubMed was the synthesis of the individual components appearing in the last table row. Asterisks denote extension of the search terms with any other additional characteristics.

**Figure 1:**
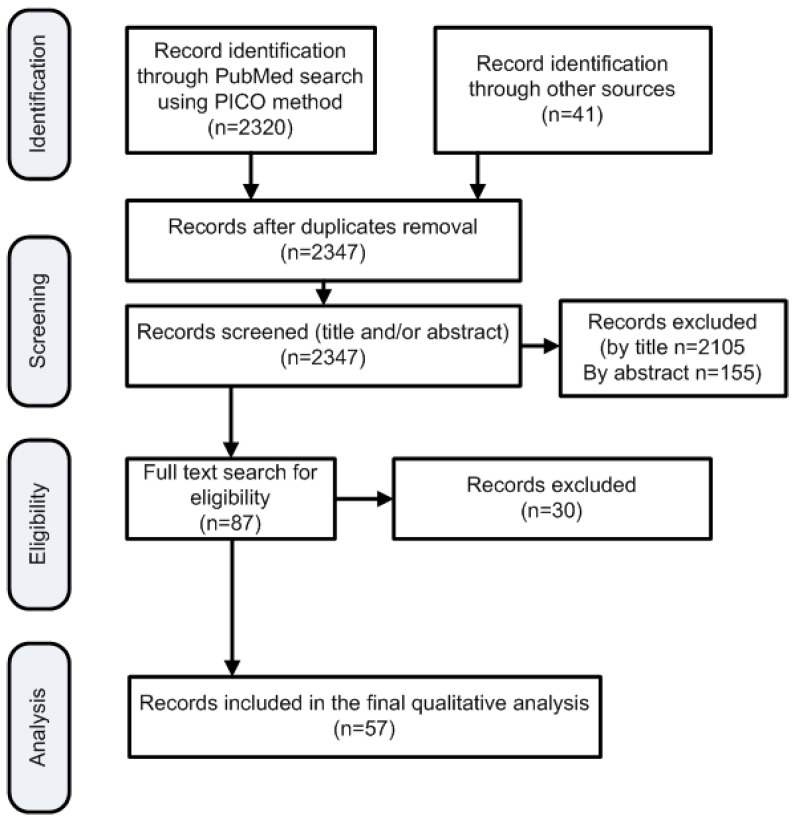
Flowchart of the search strategy according to the PRISMA framework.

**Figure 2:**
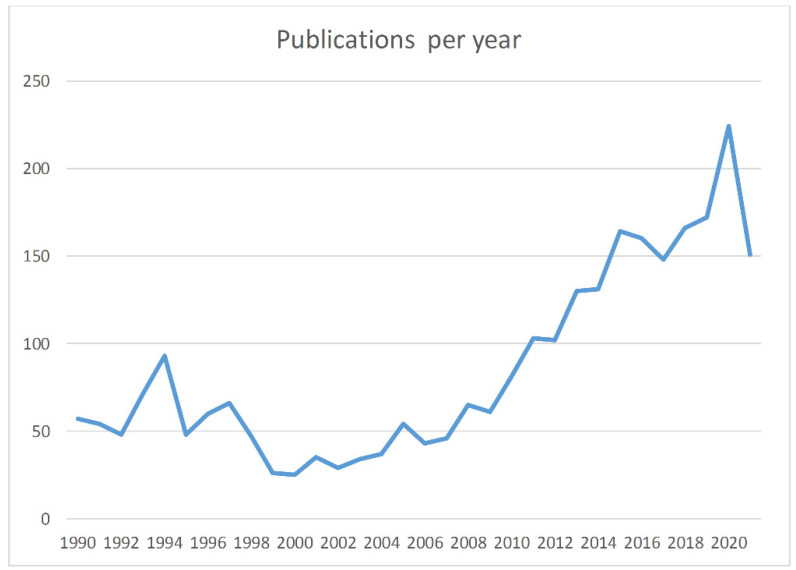
Publications by year. For 2021 data, this goes up to the end of October.

This systematic search returned 2320 publications; additional publications not found by the PICO question but known to the authors were included (n = 41) (Figure 1). Two researchers (DIM and AP) reviewed all search results independently (screening process). The review was based on titles and abstracts and irrelevant studies were included for the subsequent stage of full text review. In case of disagreements the opinion of a third researcher (TB) was requested. Other researchers participated in the data extraction stages. Figure 1 depicts the various steps of data collection and selection process and Figure 2 the initial publications for each year. Finally 57 publications were found eligible for inclusion. Notably the number of publications has an increasing trend in the last decade (Figure 2) indicative that the study subject has become more interesting to the scientific community. As no quantitative results were reported in the majority of the studies, and the focus of this review is under various conditions (i.e., hypoxia/asphyxia, ischemia/reperfusion injury of the intestinal barrier, and necrotizing enterocolitis), this study does not report meta-analysis results such as pooled effects or heterogeneity indexes, such as Cochran’s Q measure or the I^2^. However, in order to ensure the quality of the included studies, articles published in PubMed listed journals were used, but conference proceedings were avoided.

## 3. Results

### 3.1. Microbial Translocation in Hypoxic/Asphyxiating Conditions

The transition from intrauterine to extrauterine life usually occurs without problems. However, 10% of newborns need support in this process, while 1% require specialized intervention [16]. Perinatal asphyxia is defined as the lack of oxygen that may occur around delivery, leading to a reduction in the diffusion of oxygen into multiple organs and tissues. It is one of the leading causes of neonatal mortality in the first week of life. Various perinatal events may cause perinatal asphyxia. According to the World Health Organization (WHO), about four million newborns worldwide develop perinatal asphyxia each year, and one million babies die from it. These numbers correspond to 38% of all deaths of children under 5 years of age. In low-income countries, 23% of all neonatal deaths are attributed to perinatal asphyxia [17,18].

Asphyxia and hypoxia are two similar terms which are used in physiology to describe an inadequate supply of oxygen to cells and tissues. The main difference between asphyxia and hypoxia is that asphyxia is caused by an injury or obstruction of the airway passages whereas hypoxia is caused by insufficient delivery, uptake or utilization of oxygen by the body’s tissues. The clinical definition of neonatal hypoxic ischemic injury is “asphyxia of the umbilical blood supply to the human fetus occurring at 36 gestational weeks or later”. Neonatal hypoxia ischemia is synonymous with hypoxic-ischemic encephalopathy occurring in the term infant. The diagnostic criteria for neonatal hypoxic ischemia are based on a set of markers demonstrated to correlate with clinical outcome. These include 5-min Apgar score of less than 5, need for delivery room intubation or CPR, umbilical cord arterial pH less than 7.00 and abnormal neurological signs, such as hypotonic muscles or lack of sucking reflex [19]. Asphyxia causes disturbance in gas exchange, resulting in hypoxemia and hypercapnia. The combination of reduced oxygen supply (hypoxia) and reduced blood supply (ischemia) leads to dysfunction of all neonatal organs, such as the myocardium, lungs and GIT, and causes nerve cell death and brain damage. The pathogenetic mechanisms are still not completely understood and there is no cure considered to be gold standard. Metabolomics technology has been able to describe perinatal pathological conditions and to demonstrate useful means of monitoring, evaluating, and identifying potential biomarkers associated with asphyxia episodes [20,21].

An in vivo study in a murine model of obstructive sleep apnea, published in 2015, investigated if intermittent hypoxia, which resembles one of the hallmarks of obstructive sleep apnea, leads to modified fecal microbiome; a higher occurrence of Firmicutes and a smaller occurrence of Bacteroidetes and Proteobacteria phyla than controls was reported. Fecal microbiota composition and diversity were altered as a consequence of intermittent hypoxia, suggesting that physiological interplays between host and gut microbiota could be deregulated in similar situations [22].

Zhdanov et al., in 2016, studied in rats the effect of reduced tissue oxygenation on inflammation of the intestinal mucosa. Mice with colitis had increased inflammatory markers and increased measured oxygen in the upper layers of the mucosa, without affecting the architecture of the epithelium, but with concomitant partial activation of Hypoxia Induced Factor 1 and adverse effects on pyruvate dehydrogenase, suggestive of reduced mitochondrial respiration. Colonic inflammation is linked to decreased tissue oxygenation, and significantly affects gut homeostasis. Even though there is crosstalk between O_2_ consumption and supply in the inflamed tissues, the mechanisms are not fully understood. The methods used in this animal model demonstrated new horizons for the study of human diseases associated with hypoxia and inflammatory response of the intestinal mucosa [23]. Table 2 summarizes studies relevant to asphyxia and microbial translocation.

### 3.2. The Impact of Ischemia/Reperfusion(I/R) Injury on the Intestinal Barrier Function

Küçükaydin et al. investigated bacterial transposition after experimental ischemic damage and reperfusion of the intestinal mucosa in rats, using blood samples from the superior mesenteric artery and portal vein as well as tissue fragments from the final ileum and mesenteric lymph nodes [23]. Polymerase Chain Reaction in these samples showed genetic material of *E. coli*, and histological studies of the same samples suggested that intestinal ischemia-reperfusion damage may result in bacterial transmission. Several studies, in in vivo model systems, demonstrated that I/R can increase intestinal mucosal permeability, further bacterial translocation, and trigger gut cytokine production. Despite the cellular heterogeneity of the gut, nonetheless the direct effects of hypoxia/reoxygenation on intestinal epithelial cells was investigated in an in vitro model. Increased monolayer permeability to phenol red, increased E. coli bacterial translocation, and decreased transepithelial electrical resistance, showed that hypoxia/reoxygenation can directly impair cellular function [24].

Sun et al. suggested that both endothelial and epithelial barrier integrity is harmed in the early phase after I/R, and that the epithelial barrier more adequately regulates macromolecular leak compared with the endothelial barrier. I/R impairs the intestinal barrier by causing tissue hypoxia and by activating the phagocytic system and irritating barrier damage, which finally may result in bacterial translocation and remote organ dysfunction [25].

More recently, a team of investigators interested in the relationship between hypoxia and I/R injury looked for the occurrence of bacterial translocation after cardiac arrest, followed or not by successful resuscitation, a field that has not yet been adequately studied. Gkiza et al. studied two groups of pig, where in the first only minimal aseptic interventions were done, while in the second ventricular fibrillation and finally obstruction was performed. The researchers advocated the existence of bacterial transmission through the mechanism of intestinal I/R damage due to cardiac arrest and they also suggested, for the first time, that cardiopulmonary arrest may lead to systemic inflammation soon after successful resuscitation [26] (Table 3).

### 3.3. Necrotizing Enterocolitis and Translocation

Table 4 summarizes the published work relevant to NEC and bacterial translocation. The GIT is particularly vulnerable to ischemic damage. Even short periods of ischemia may cause significant local tissue damage. Fetal hypoxia and perinatal asphyxia act by reducing bowel motility, especially in preterm infants. Previous studies in animal model systems showed that oxygen administration during airway management prevents hypoxemia, intestinal harm, and bacterial translocation [28]. As it becomes obvious from recent literature, ischemic preconditioning seems to be beneficial for the human heart and the liver. Prospective controlled studies in humans, including ischemic preconditioning of the intestine, are lacking [29].

Necrotizing enterocolitis is an inflammatory bowel disease that affects newborns, especially premature ones, with 15–30% mortality and 10–50% long-term morbidity [30]. Survivors suffer from short-term gastrointestinal complications (feeding difficulties), as well as long-term complications (reduced growth, possible liver disease due to parenteral nutrition, and short bowel syndrome) [30,31]. Less than 10% of all NEC cases involve full-term newborns [32]. The gestational age is inversely proportional to the time of onset of NEC, i.e., premature infants develop the disease later in life, while full-term babies appear to get sick earlier. The pathogenesis remains unclear, but it is believed that in premature babies, bacterial invasion and activation of the inflammatory process occur with possible subsequent cell necrosis, due to dysfunction or immaturity of the intestinal barrier. In full term infants, different clinical conditions were identified as possible risk factors, including perinatal hypoxia/asphyxia, certain congenital heart diseases, polycythemia/thrombotic conditions, endocrine diseases and perinatal sepsis [33].

The distal ileum and the proximal colon, are the most commonly affected parts of the intestine in neonates with necrotizing enterocolitis [34]. Current data suggest that intestinal microbial dysbiosis precedes NEC in premature infants. However, further research, with large prospective studies and standard methodology is needed to understand the significance of the changes that occur in the microbiome during the early postnatal period, in order to assess the reproducibility of the available data on the issue [35,36].

The pathophysiology of NEC is multifactorial. Ischemic injury of the intestinal mucosa is caused by the “diving reflex”, where peripheral vasoconstriction and redistribution of blood from the non-vital to vital organs such as heart, brain and adrenal glands occur in response to the drop in blood pressure during asphyxia. This type of ischemic bowel injury is one of the pathogenetic mechanisms of NEC, secondary to infection or asphyxia. Due to hypoxic injury, production of nitric oxide, which is necessary to maintain the proper perfusion of the intestine, is reduced, with a consequent reduction in the resistance of the intestinal vessels [37,38]. The increased bacterial translocation by the disturbed intestinal epithelium activates endogenous signaling, such as the platelet activating factor and the tissue necrosis membrane, inducing the cataract of inflammation. What remains to be clarified is the exact point where the various aforementioned clinical factors play a role. For example, is intestinal feeding, bacteria, or hypoxic damage simply the initiator of this pathway? Studies have focused on these factors, as they may be involved in the etiology of NEC [39].

The GIT is supported by a rich and multiplex underlying vasculature. As a result, the intestinal epithelial cell layer is susceptible to damage associated with decreased blood flow. The resulting hypoxia is a consequence of both diminished perfusion and increased metabolism within the mucosa. The metabolic shift may result in “cytopathic hypoxia,” a type of mitochondrial dysfunction which leads to reduced intracellular oxygen and ATP availability. Even though, this type of damage constitutes a risk to the epithelial function by restricting harmful luminal entities, recent data have suggested that the intestinal epithelium is equipped with hypoxia-inducible adaptive mechanisms, which sustain barrier function under conditions of such a dysfunction. Compared with other mucosal surfaces, intestinal epithelial cells seem to be uniquely resistant to disruption by hypoxia. Such observations may relate to the fact that intestinal epithelial cells are conditioned to a lower pO2 than other tissues [40].

Despite the fact that there is strong correlation between barrier breakdown and bacterial translocation, the molecular mechanisms of bacterial translocation from the lumen of the GIT to the bloodstream are not well understood. Although there is an increase in translocated bacteria with hypoxia, most evidence suggests that overall integrity of the intestinal epithelium remains intact, even in relatively severe hypoxia. This finding may indicate that bacterial translocation during intestinal inflammation is a consequence of increased transcellular bacterial movement, rather than a breakdown of epithelial integrity [41].

In a recent study of a pig cardiopulmonary resuscitation model, the authors attempted to highlight the metabolic profile in plasma samples from asphyxiated animals and animals with ventricular fibrillation. The metabolic profile of the two groups differed significantly during cardiac arrest and during the resuscitation phase. Animals with the worst outcome had overproduction of the electro-coenzyme A (Krebs cycle), suggesting a possible prognostic role for this metabolite [42]. The intestinal mucosa becomes ‘injured’, its structure alters, the intestinal barrier is weakened, microbial populations move through the disturbed intestinal barrier, and mediators of inflammation are released. The corresponding part of the GIT ‘dies’, while the initial local damage leads to a generalized response of the body as various microorganisms and endotoxins travel through the blood to peripheral tissues and organs (bacterial translocation), with potentially life-threatening consequences, such as sepsis and multiple organ dysfunction syndrome. The various bacterial strains enter the systemic circulation via the intestinal venous system and the portal vein, or through the intestinal lymphatic route [43,44,45].

**Table 4:** The relationship between gut microbiota, intestinal barrier function and microbial translocation and necrotizing enterocolitis.

Authors	Year	Topic	Outcome
Ali Nayci et al. [28]	2006	Oxygen supplementation during airway instrumentation improves intestinal barrier dysfunction	Oxygen prevents hypoxemia, intestinal damage, and bacterial translocation
Mallick et al.[29]	2004	I/R injury of the intestine and protective strategies against injury	Prospective controlled studies in humans involving ischemic preconditioning of the intestine are lacking
Berman et al. [31]	2011	NEC: an update	Most frequent long-term complications include short bowel syndrome, abnormal growth, neurodevelopmental delay.
Ostlie et al. [32]	2003	NEC in full-term infants	10% of cases of NEC are full term infants
Short et al. [33]	2014	Late onset of NEC in the full-term infant is associated with increased mortality: results from a two-center analysis	The gestational age is inversely proportional to the time of onset of NEC. Possible risk factors include perinatal hypoxia/asphyxia, congenital heart diseases etc.
Iben et al. [34]	2011	NEC	The distal ileum and the proximal colon, are the most commonly affected in NEC
Patole et al. [35]	2017	Microbiota and NEC	Microbial dysbiosis has been implicated in pathogenesis of NEC
Pammi et al. [36]	2017	Intestinal dysbiosis in preterm infants preceding NEC: a systematic review and meta-analysis	Further research is needed in order to assess the reproducibility of the available data on the issue
Grishin et al. [37]	2016	Roles of nitric oxide and intestinal microbiota in the pathogenesis of NEC	Nitric oxide plays a prominent role in the intestinal barrier damage by inducing enterocyte apoptosis and inhibiting the epithelial restitution processes
Stevenson et al. [38]	2006	Historical perspectives: NEC an inherited or acquired condition?	Intestinal mucosa ischemic injury caused by the “diving reflex” occurs in response to the decrease of blood pressure during perinatal asphyxia
Keely et al. [39]	2010	Hypoxia-inducible factor-dependent regulation of platelet-activating factor receptor as a route for gram-positive bacterial translocation across epithelia	It remains to be determined whether HIF-mediated, PAFr-dependent bacterial translocation represents a physiological clearance mechanism or rather serves as a pathophysiologic mechanism whereby bacteria exploit PAFr as a route of entry
Glover et al. [40]	2016	Oxygen metabolism and barrier regulation in the intestinal mucosa	In the intestine, baseline pO2 levels are uniquely low due to counter-current blood flow and the presence of large numbers of bacteria and this mechanism contributes to the gut mucosa homeostasis
Tugtekin et al. [41]	2001	Increased ileal-mucosal-arterial PCO2 gap is associated with impaired villus microcirculation in endotoxic pigs	Increased ileal-mucosal-arterial delta PCO2 during porcine endotoxemia is related to impaired villus microcirculation
Varvarousis et al. [42]	2017	Metabolomics profiling reveals different patterns in an animal model of asphyxial and dysrhythmic cardiac arrest	Succinate overproduction was observed in the animals with the worse outcome, suggesting a potential prognostic role for this metabolite
Lim et al. [43]	2015	Pathogenesis of NEC	Opportunistic pathogens breach the gut barrier and incite an inflammatory response that leads to overproduction of inflammatory mediators which exacerbate the initial mucosal injury and also suppress the intestinal repair mechanisms
Hackam et al. [44]	2013	Mechanisms of gut barrier failure in the pathogenesis of NEC: Toll-like receptors throw the switch	Activation of the receptor for bacterial endotoxin, TLR4, is required for the development of intestinal barrier failure leading to NEC
Patel et al. [45]	2015	Intestinal microbiota and its relationship with NEC	Shifting the balance of intestinal microbiota from a pathogenic to protective complement of bacteria can protect the gut from inflammation and subsequent injury that leads to NEC

### 3.4. Bacterial Endotoxins

Nine publications related to bacterial endotoxins were identified (Table 5). The term “endotoxin” is synonymous, and is used interchangeably with, the term lipopolysaccharide (LPS). LPS, which comes exclusively from Gram-negative bacteria, has been the gold standard in the study of the basic mechanisms of sepsis for years [45]. According to the U.S. National Library of Medicine, using the term endotoxin we describe “toxins closely linked to the cytoplasm or cell wall of microorganisms, which are not readily distributed in the culture medium but are released by the cell solution.” Endotoxins cause fever and, in larger doses, shock and death. They also provoke an inflammatory response through interaction with high-affinity receptors on leukocytes [45]. The increase in plasma LPS levels disrupts the intestinal mucosal barrier and the transfer of bacteria and their components into the circulation. High levels of LPS are seen in systemic sepsis and in NEC. Intestinal barrier function is affected through different pathways, including direct effects on the repression of intestinal restitution, and indirect effects such as by promoting the release of signaling molecules from enterocytes, including NO and interferon (IFN)-γ. Endotoxin signaling in enterocytes is mainly mediated by a receptor called toll-like receptor 4 (TLR4) and myeloid differentiation factor 2 (MD-2) [46,47]. Wolfs et al., using immunochemistry in the normal and inflamed ileum of neonates and adults, reported that the absence of MD-2 in the immature neonatal gut suggests impaired LPS sensing, which could predispose neonates to NEC upon microbial colonization of the immature intestine. The obvious expression of MD-2 by Paneth cells supports the censorious idea that these cells respond to luminal bacterial products in order to maintain homeostasis with the intestinal microbiota in vivo [48].

Systemic stress causes breakdown in the intestinal mucosal barrier, leading to translocation of bacteria and endotoxin and the initiation of a signaling response within the enterocyte. Enterocyte signaling plays a principle role in the pathogenesis of NEC by the following mechanisms: (1) Local villus enterocytes produce nitric oxide, which increases in enterocyte apoptosis and impaired multiplication, (2) Translocation of endotoxin causes a Phosphoinositide 3-kinase -dependent activation of Ras homolog family member A-GTPase within the enterocyte leading to decreased enterocyte migration and impaired restitution, (3) Dysregulated by endotoxin, sodium–proton exchange makes the enterocyte monolayer more susceptible to damage in an acidic microenvironment characteristic of systemic sepsis, (4) Endotoxin, finally, is associated with a mitogen-activated protein kinase p38-dependent release of the pro-inflammatory molecule Cyclooxygenase-2 by the enterocyte, which intensifies systemic inflammatory response [49].

Sodium/proton exchangers (NHE), present at the basolateral and apical surfaces of enterocytes, are essential for the preservation of enterocyte activity during extracellular acidosis. Necrotizing enterocolitis is characterized by systemic hypoperfusion, metabolic acidosis, and the apical to basolateral translocation of LPS. Cetin, Dunklebarger et al. hypothesized that LPS differentially impairs NHE activity at the basolateral and apical zones of enterocytes, leading to cellular acidification, and investigated the mechanisms involved. Experimental NEC was induced in newborn rats using a combination of gavage feeds and hypoxia. Results of Western blot analysis and confocal microscopy in the presence or absence of LPS suggest that LPS selectively diminishes basolateral NHE1 but not apical NHE3, leading to cytoplasmic acidification during extracellular acidosis. This could damage enterocyte function after LPS translocation and also suggests a mechanism leading to barrier disruption in NEC [50].

Experimental necrotizing enterocolitis (NEC) is characterized by circulating LPS and impaired enterocyte migration. Cetin, Dunklebarger et al. reported that enterocyte migration is inhibited by LPS through increased expression and function of alpha 3- and beta 1-integrins and suggested that modulation of enterocyte migration via integrins may provide novel insights into the pathogenesis of NEC, in which intestinal restitution is impaired [51].

In the course of several years, research has tried to crack the case between hypoxia or asphyxia and LPS at a molecular level. Corcoran & O’Neill described the induction of Hypoxia Induced Factor 1a (HIF1a) by LPS-activated macrophages, which is of fundamental importance in glycolysis and the induction of pro-inflammatory genes, particularly that of interleukin-1 (IL-1). LPS seems to affect other key points in the inflammatory sequelae, promoting or inhibiting other metabolites [52]. Yet these mechanisms continue to be poorly understood.

## 4. Discussion

Data of bacteria involved in the pathogenesis of NEC is limited by the infant’s fragility, the restriction of analysis to feces, the use of culture-based methods, and the lack of clinically-relevant animal models.

Perinatal asphyxia is a complex phenomenon that affects the health status of mammals at multiple levels, and although researchers have been largely concerned over recent years, there are not yet enough studies of animal models that examine the association between microbial translocation induced by hypoxemia and tissue damage as a result of ischemia or ischemia and subsequent reperfusion.

There is also insufficient data from clinical or laboratory tests related to the subject under study. There are studies in animal models, mainly rats, mice, rabbits, quails, piglets, and non-human primates, but there is still lack of information about perinatal and neonatal age in humans. These studies provide important knowledge, but leave many questions unanswered about the pathogenetic mechanisms of life-threatening conditions associated with perinatal asphyxia, especially at the microbial level. Most research data focus on the effect of hypoxia/asphyxia on the brain or gut–brain axis, with an extensive analysis of the pathophysiology of ischemic brain injury. In contrast, data on the effect of these conditions on other organs, such as GIT, liver, spleen, lungs and systemic inflammation, are scarce.

Due to biochemical complexities beyond the scope of studies in single-cell cultures, animal models are essential to understand the mechanisms involved in conditions that resemble the pathophysiology of NEC and the effects of inflammation on the immature intestinal tract [55,56,57], as shown in Table 6.

A fundamental approach to this is the use of in vivo experimental neonatal rodents and pigs. Much of the experimental evidence derives from models in rodents, and show the protective effect of breast milk and the role of specific molecular mechanisms involved in premature innate immune and intestinal injury response. This type of model tests how genetic disruption of specific genes alters the NEC phenotype. More recently, pigs have emerged as an animal model of NEC and are used to establish the role of bacterial colonization, prematurity, parenteral nutrition and antibiotic therapy [58]. The pig is the closest animal to the human, in terms of both anatomy and physiology, and therefore it allows simulation and projection of findings for humans. The hemodynamic parameters in neonatal piglets (heart rate and blood pressure) are also comparable to those of humans. In addition, the pathophysiology of the response to neonatal asphyxia is similar, which consolidates the role of the pig as an experimental animal in the study of the effects of the latter. Furthermore, the similarities between pigs and humans extend to the size of various organs and immune mechanisms [59].

The purpose of this review was mainly to highlight: (a) the effect of a systemic situation (asphyxia) on a system/tissue and the human body as a whole (due to the bacterial translocation, if any); (b) the study of existing data on physiology–pathophysiology. Given the complexity of the effect of asphyxia and the constant interaction between cells and systems of a living multicellular organism, mediated by endogenous chemicals and signaling molecules, it would be virtually impossible for an in vitro model to offer conclusions.

The major limitation of this study is the lack of a systematic analysis of the studies’ quality, and notably the majority of the papers involved in this study are reviews (non-systematic). Moreover, there are numerous studies presenting results of in vitro experiments and experiments on animal models, which are considered of low evidence in the evidence based medicine pyramid. In Appendix A studies involved in this systematic review are presented along with the study type and the level of evidence according to the evidence based medicine pyramid [61]. Another issue of our study, and in general studies of this type, due to ethical reasons, is that the vast majority are either in vitro or based on animal models, and therefore are certainly pre-clinical and, moreover, are considered of low evidential quality. Notably, among the analyzed studies, only one was on human subjects [50]. To our knowledge, this is the first review performed in a systematic manner.

## 5. Conclusions

In conclusion, it is clear that further scientific studies using multicellular organisms are needed. Each animal has distinct advantages and disadvantages related to its viability, body size, genetic differences, and cost. The choice of the kind of animal model is strongly influenced by the scientific question that the researchers seek to answer [60]. As analyzed above, pigs seem to be a sufficient option in this case, although no model may perfectly mimic human diseases such as NEC. Thus, it will be possible partially to generalize our conclusions on the human body, where communication between different cell and tissue types is complex and cannot be artificially simulated at present.

## Figures and Tables

**Table 1:** Individual PICO Components.

Component	Query
P(Patient, Problem or Population)	((infant*[Title/Abstract] or infant*[MeSH Terms]) OR (neonat*[Title/Abstract] or neonat*[MeSH Terms]) OR (newborn[Title/Abstract] or newborn[MeSH Terms])) OR ((animal*[Title/Abstract] or animal*[MeSH Terms]) OR (perinat*[Title/Abstract] or perinat*[MeSH Terms]) OR (child*[Title/Abstract] or child*[MeSH Terms]) OR (model*[Title/Abstract] or model*[MeSH Terms]) OR (piglet[Title/Abstract] or piglet[MeSH Terms]))
I (Intervention)	((asphyxia[Title/Abstract] or asphyxia[MeSH Terms]) OR (hypoxia[Title/Abstract] or hypoxia[MeSH Terms]) OR (necrot* enterocolitis[Title/Abstract] or necroti* enterocolitis[MeSH Terms]) OR (NEC[Title/Abstract] or NEC[MeSH Terms]))
C(Comparison, control or comparator)	Not used
O (Outcome)	((gastro*[Title/Abstract] or gastro[MeSH Terms]) OR (intestin*[Title/Abstract] or intestin*[MeSH Terms]) OR (gut[Title/Abstract] or gut[MeSH Terms])) AND ((microb*[Title/Abstract] or microb*[MeSH Terms]) OR (bacter*[Title/Abstract] or bacter*[MeSH Terms]) OR (translocation[Title/Abstract] or translocation[MeSH Terms]) OR (toxine*[Title/Abstract] or toxine*[MeSH Terms]))
Τ (Time)	(“1990/01/01” [Publication Date]: “3000” [Publication Date])
PICO Question	((infant*[Title/Abstract] or infant*[MeSH Terms]) OR (neonat*[Title/Abstract] or neonat*[MeSH Terms]) OR (newborn[Title/Abstract] or newborn[MeSH Terms])) OR ((animal*[Title/Abstract] or animal*[MeSH Terms]) OR (perinat*[Title/Abstract] or perinat*[MeSH Terms]) OR (child*[Title/Abstract] or child*[MeSH Terms]) OR (model*[Title/Abstract] or model*[MeSH Terms]) OR (piglet[Title/Abstract] or piglet[MeSH Terms])) AND ((asphyxia[Title/Abstract] or asphyxia[MeSH Terms]) OR (hypoxia[Title/Abstract] or hypoxia[MeSH Terms]) OR (necrot* enterocolitis[Title/Abstract] or necroti* enterocolitis[MeSH Terms]) OR (NEC[Title/Abstract] or NEC[MeSH Terms])) AND AND((gastro*[Title/Abstract] or gastro[MeSH Terms]) OR (intestin*[Title/Abstract] or intestin*[MeSH Terms]) OR (gut[Title/Abstract] or gut[MeSH Terms])) AND ((microb*[Title/Abstract] or microb*[MeSH Terms]) OR (bacter*[Title/Abstract] or bacter*[MeSH Terms]) OR (translocation[Title/Abstract] or translocation[MeSH Terms]) OR (toxine*[Title/Abstract] or toxine*[MeSH Terms])) AND (“1990/01/01”[Publication Date]: “3000”[Publication Date])

**Table 2:** Systemic research results for Microbial Translocation in Neonatal Hypoxic/Asphyxiating conditions.

Authors	Year	Topic	Outcome
International Liaison Committee on Resuscitation [16]	2005	International Consensus on Cardiopulmonary Resuscitation and Emergency Cardiovascular Care Science with Treatment Recommendations. Part 7: Neonatal resuscitation	The majority of newborn infants do not need specialized medical intervention peripartum, but the large number of births worldwide means that many infants require some resuscitation
Fattuoni et al. [17]	2015	Perinatal Asphyxia: A Review from a Metabolomics Perspective	Oxygen deprivation that occurs around the time of birth, caused by several perinatal events, affects one million neonates worldwide per year, causing even death
Aslam et al. [18]	2014	Risk factors of birth asphyxia	Birth asphyxia leads to decreased oxygen perfusion and malfunction in vital organs
Antonucci et al. [20]	2014	Perinatal asphyxia in the term newborn	Despite the advances in perinatal care, asphyxia remains a severe condition leading to significant mortality and morbidity
Rainaldi et al. [21]	2016	Pathophysiology of Birth Asphyxia	Asphyxia generally results from interruption of placental blood flow with resultant fetal hypoxia, hypercarbia, and acidosis
Moreno et al. [22]	2016	Sleep recovery mimicking treatment of sleep apnea does not reverse intermittent hypoxic-induced dysbiosis and low-grade endotoxemia in mice	Gut microbiota composition and circulating endotoxemia remain negatively altered after a post-intermittent hypoxia normoxic period in mice with obstructive sleep apnea

**Table 3:** The intestinal barrier function and I/R injury.

Authors	Year	Topic	Outcome
Küçükaydin et al. [24]	2000	Detection of intestinal bacterial translocation in subclinical I/R using the polymerase chain reaction (PCR) technique	PCR detecting microbial DNA, showed that subclinical intestinal I/R injury in rats results in bacterial translocation
Xu et al. [25]	1999	The effect of hypoxia/reoxygenation on the cellular function of intestinal epithelial cells	Hypoxia/reoxygenation can directly impair cellular function
Sun et al. [26]	2000	Phagocytic and intestinal endothelial and epithelial barrier function during the early stage of small intestinal I/R injury	Endothelial and epithelial barrier integrity is harmed in the early phase after I/R
Gkiza et al. [27]	2012	Isolation of aerobic bacteria in internal specimens from domesticated pigs used in biomedical research and the association with bacterial translocation.	There is bacterial transmission caused by intestinal I/R damage due to cardiac arrest

**Table 5:** Bacterial lipopolysaccharides (LPS/Endotoxins) and their role in the inflammatory pathway of NEC.

Authors	Year	Topic	Outcome
Beutler et al. [46]	2003	Innate immune sensing and its roots: the story of endotoxin	Host sensors named Toll-like receptors take part in chemical, biological and genetic analyses centred on a bacterial poison and termed endotoxin
Jiang et al. [47]	1995	Kinetics of endotoxin and tumor necrosis factor appearance in portal and systemic circulation after hemorrhagic shock in rats	Hemorrhagic shock may lead to early bacterial translocation in the intestinal wall and transient access of gut-derived LPS and LPS-induced mediators via the portal circulation
Yao et al. [48]	1995	Pathogenesis of hemorrhage-induced bacteria-endotoxin translocation in rats: effects of recombinant bactericidal-increasing protein (rBPI21)	Hemorrhagic shock may lead to bacterial/endotoxin translocation with concomitant TNF formation, and endogenous endotoxemia may play an important role in the pathogenesis of multiple-organ failure after shock and trauma
Anand et al. [49]	2007	The role of the intestinal barrier in the pathogenesis of necrotizing enterocolitis	Disruption in barrier function and bacterial translocation are of particular concern to the newborn patient, due to the risk of intestinal inflammation
Wolfs et al. [50]	2010	Localization of the lipopolysaccharide recognition complex in the human healthy and inflamed premature and adult gut	The absence of MD-2 in the immature neonatal gut suggests impaired LPS sensing, predisposing to NEC upon microbial colonization of the immature intestine
Hackam et al. [51]	2005	Disordered enterocyte signaling and intestinal barrier dysfunction in the pathogenesis of necrotizing enterocolitis	Systemic stress causes a breakdown in the intestinal mucosal barrier, which leads to translocation of bacteria and endotoxin and the initiation of a signaling response within the enterocyte
Cetin et al. [52]	2004	Endotoxin differentially modulates the basolateral and apical sodium/proton exchangers (NHE) in enterocytes	LPS selectively impairs basolateral NHE1 leading to cytoplasmic acidification during extracellular acidosis, impairing enterocyte function after translocation
Qureshi et al. [53]	2005	Increased expression and function of integrins in enterocytes by endotoxin impairs epithelial restitution	Enterocyte migration is inhibited by LPS through increased expression and function of alpha 3- and beta 1-integrins
Corcoran et al. [54]	2016	HIF1α and metabolic reprogramming in inflammation	HIF1α is induced in LPS-activated macrophages, where it is critically involved in glycolysis and the induction of pro-inflammatory genes

**Table 6:** Investigation of pathogenetic mechanisms of NEC and other common GIT diseases of neonates associated with microbiota and the use of different research methods.

Authors	Year	Topic	Outcome
Ares et al. [55]	2018	The science and necessity of using animal models in the study of necrotizing enterocolitis	Animal models are essential to understand the mechanisms involved in the pathophysiology of NEC and the effects of inflammation on the immature intestinal tract
Azcarate-Peril et al. [56]	2011	Acute necrotizing enterocolitis of preterm piglets is characterized by dysbiosis of ileal mucosa-associated bacteria	Ileal mucosa seems to be a fundamental part of GIT for investigation of dysbiosis associated with NEC
Berthe C Oosterloo et al. [57]	2014	Dual purpose use of preterm piglets as a model of pediatric GI disease	Both rodent and pig models have advantages and disadvantages as experimental models of NEC
Aroni et al. [58]	2012	An experimental model of neonatal normocapnic hypoxia and resuscitation in Landrace/ Large White piglets	Hemodynamic fluctuations at baseline during normocapnic hypoxia and reoxygenation in Landrace/Large White piglets are comparable to those in human neonates
Sangild et al. [59]	2013	The preterm pig as a model in pediatric gastroenterology	The preterm pig appears to be a translational model in pediatric gastroenterology and has provided new insights into important pediatric diseases such as NEC
Barré-Sinoussi et al. [60]	2015	Animal models are essential to biological research: issues and perspectives	Animal models have been used to address a variety of scientific questions

## Data Availability

Not applicable.

## Appendix A. Evaluation of Studies Quality/Level of Evidence According to the Evidence Based Medicine Pyramid

**Table A1:** Systemic research results on Microbial Translocation in Neonatal Hypoxic/Asphyxiating conditions.

Authors	Year	Study Type	Level of Evidence
International Liaison Committee on Resuscitation [16]	2005	Guideline	High
Fattuoni et al. [17]	2015	Review	High
Aslam et al. [18]	2014	Retrospective Case control study	Medium
Antonucci et al. [20]	2014	Review	High
Rainaldi et al. [21]	2016	Review	High
Moreno et al. [22]	2016	RCT on mice (10 and 10)	Medium

**Table A2:** Intestinal barrier function and I/R injury.

Authors	Year	Study Type	Level of Evidence
Küçükaydin et al. [24]	2000	RCT on mice (20, 20 and d20)	Medium
Xu et al. [25]	1999	In vitro study on cell cultures	Low
Sun et al. [26]	2000	RCT on mice	Medium
Gkiza et al. [27]	2012	Case control study on piglets (24 and 22)	Medium

**Table A3:** The relationship between gut microbiota, intestinal barrier function and microbial translocation and necrotizing enterocolitis.

Authors	Year	Study Type	Level of Evidence
Ali Nayci et al. [28]	2006	RCT on mice (15 20 and 20)	Medium
Mallick et al. [29]	2004	Review	High
Berman et al. [31]	2011	Seminar paper/educational	High
Ostlie et al. [32]	2003	Retrospective case control study	Medium
Short et al. [33]	2014	Retrospective review	Medium
Iben et al. [34]	2011	Book chapter	High
Patole et al. [35]	2017	Review	High
Pammi et al. [36]	2017	Systematic review and meta-analysis	High
Grishin et al. [37]	2016	Review	High
Stevenson et al. [38]	2006	Personal insight	Low
Keely et al. [39]	2010	In vitro study on cell cultures	Low
Glover et al. [40]	2016	Review	High
Tugtekin et al. [41]	2001	RCT on domestic pigs (12 with toxin and 10 without)	Medium
Varvarousis et al. [42]	2017	RCT on swine (10: asphyxial cardiac arrest and 10: ventricular fibrillation cardiac arrest)	Medium
Lim et al. [43]	2015	Review	High
Hackam et al. [44]	2013	Review (description of current research)	High
Patel et al. [45]	2015	Review	High

**Table A4:** Bacterial lipopolysaccharides (LPS/Endotoxins) and their role in the inflammatory pathway of NEC.

Authors	Year	Study Type	Level of Evidence
Beutler et al. [46]	2003	Review	High
Jiang et al. [47]	1995	Experiment on 37 rats	Low
Yao et al. [48]	1995	RCT on rats	Medium
Anand et al. [49]	2007	Review	High
Wolfs et al. [50]	2010	Specimens obtained from the ileum of infants and adults and stored tissue material	Medium
Hackam et al. [51]	2005	Review	High
Cetin et al. [52]	2004	Cell culture	Low
Qureshi et al. [53]	2005	Experiment on rats	Low
Corcoran et al. [54]	2016	Review	High

**Table A5:** Investigation of pathogenetic mechanisms of NEC and other common GIT diseases of neonates associated with microbiota and the use of different research methods.

Authors	Year	Study Type	Level of Evidence
Ares et al. [55]	2018	Review on animal models	High
Azcarate-Peril et al. [56]	2011	Study on 23 piglets	High
Berthe C Oosterloo et al. [57]	2014	Review on piglet models	High
Aroni et al. [58]	2012	Study on 10 piglets	Low
Sangild et al. [59]	2013	Review on piglet animal models	High
Barré-Sinoussi et al. [60]	2015	Opinion	Low
